# Association between pre-pregnancy weight status and dietary patterns during pregnancy: results from the Japan Environment and Children’s Study

**DOI:** 10.1017/S1368980023000770

**Published:** 2023-09

**Authors:** Kazue Ishitsuka, Kiwako Yamamoto-Hanada, Hidetoshi Mezawa, Mayako Saito-Abe, Hatoko Sasaki, Minaho Nishizato, Miori Sato, Yukihiro Ohya, Japan Environment and Children’s Study Group

**Affiliations:** Medical Support Center of JECS Study, National Center for Child Health and Development, Tokyo 1578535, Japan

**Keywords:** Dietary pattern, Pregnancy, Pre-pregnancy weight status, Principle component analysis

## Abstract

**Objective:**

Pre-pregnancy weight status is related to offspring health and may influence dietary patterns during pregnancy. We aimed to evaluate the link between pre-pregnancy weight status and dietary patterns during pregnancy.

**Design:**

Dietary data were collected using a FFQ during middle or late pregnancy. Dietary patterns were identified using principal component analysis. Anthropometric data were extracted from medical charts. Multiple linear regression was used to assess associations between pre-pregnancy weight status (severely or moderately underweight, mildly underweight, normal weight, overweight and obese) and dietary patterns during pregnancy after adjusting for socio-demographic characteristics.

**Setting:**

Nationwide Japan.

**Participants:**

Pregnant Japanese women enrolled in the Japan Environment and Children’s Study, a prospective birth cohort study (*n* 90 765).

**Results:**

We identified three dietary patterns. Compared with women with pre-pregnancy normal weight, those with pre-pregnancy obesity were less likely to habitually consume ‘fruits and vegetables’ pattern (coefficient, –0·18; 95 % CI, –0·21, –0·14) and ‘confectionery’ pattern (coefficient, –0·18; 95 % CI, –0·21, –0·14) and more likely to consume ‘white rice and soy products’ pattern (coefficient, 0·08; 95 % CI, 0·04, 0·11), and those with severely or moderately pre-pregnancy underweight were more likely to consume ‘confectionery’ pattern (coefficient, 0·06; 95 % CI, 0·03, 0·09) during pregnancy, after adjusting for confounders.

**Conclusion:**

We found that moderately and severely pre-pregnancy underweight women and those with obesity had unhealthy dietary patterns compared to those with pre-pregnancy normal weight. Our findings suggest that prenatal dietary advice is important and should be based on the pre-pregnancy weight status.

Preconception care is crucial for maternal and child health. Accumulating evidence indicates that a woman’s pre-pregnancy weight status is related to women’s and child health^(1–4)^. Pre-pregnancy overweight or obese women have an increased risk of macrosomia, neural tube defects and cardiovascular anomalies^(3,4)^. Dietary intake may mediate the associations between pre-pregnancy weight status and child outcomes^(5–10)^. Some studies have shown that pregnant women with obesity are less likely to adhere to a health-conscious dietary pattern, which can result in unfavourable child outcomes^(5–8)^.

Pre-pregnancy underweight pregnant women have an increased risk of fetal growth failure and preterm delivery^(1,2)^. The growth failure of the fetus in underweight pregnant women may be due to inadequate macro- and micro-nutrients, which may also lead to CVD in later life^(9,10)^.

Thinness is considered an ideal beauty standard for women of reproductive age^(11,12)^. The desire for thinness, which leads to inappropriate attempts to lose weight in an unhealthy way, sometimes results in women being underweight^(13)^. A European study showed that underweight women were more likely to skip meals than normal-weight women, resulting in inadequate nutrient intake^(14)^. In addition, women who attempt to lose weight may avoid foods that they believe might make them fat. Therefore, understanding dietary patterns in underweight women can help establish strategies for nutritional recommendations. Furthermore, the traditional single-nutrient approach in some studies overlooks collinearity among nutrients^(15,16)^. Thus, examining dietary patterns instead can better indicate a person’s overall diet and may help elucidate the interactive and synergistic effects of nutrients^(15)^.

Notably, Japan has a high prevalence of underweight women of reproductive age, and many women desire to be thin^(17)^. However, to the best of our knowledge, no study has investigated the dietary patterns in pregnant women according to the pre-pregnancy weight status in Japan. A large Japanese cohort study has obtained information on dietary intake at the second and third trimesters.

This study aimed to investigate the association of pre-pregnancy weight status with dietary patterns during pregnancy using data from a nationwide Japanese birth cohort study. We hypothesised that both pre-pregnancy underweight and overweight pregnant women follow nutrient-poor dietary patterns than women with normal pre-pregnancy weight.

## Materials and methods

### Study participants

This study was a secondary analysis based on data gathered from the Japan Environment and Children’s Study (JECS), a nationwide birth cohort study. The design of the JECS has been described in detail previously^(18,19)^. Briefly, the main objective of the JECS was to investigate the influence of environmental chemical exposures on child health and development. Women were recruited throughout pregnancy (mean gestational age at recruitment: 13·2 weeks, sd 8·4 weeks). Dietary intake during pregnancy was assessed using a FFQ, which was administered during middle or late pregnancy (mean gestational age: 27·9 weeks, sd 6·5 weeks).

**Fig. 1:**
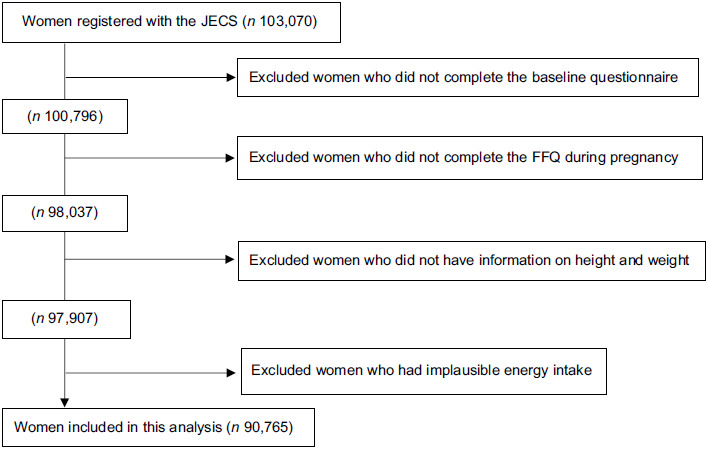
Flow diagram of study participants. Abbreviation: FFQ, food frequency questionnaire; JECS, the Japan Environment and Children’s study

Pregnant women were recruited at the clinic during prenatal checkups or at local government offices, where they were requested to register their pregnancy after learning about their conception, in regional centres located in urban and rural areas across Japan. The eligibility criteria were pregnant women who lived in the study area, and whose expected delivery date was between 2011 and 2014. The number of eligible women in each study area ranged from 130 000 to 600 000. Pregnant women were continuously recruited until the number of total study participants in all areas reached over 100 000. Women who could not use the Japanese language to complete the self-administered questionnaires were excluded from the JECS. A total of 103 070 pregnancies were registered in JECS (Fig. 1). Of these, 100 796 answered the baseline questionnaire for JECS. For this analysis, we included women who answered the FFQ to assess their dietary intake during pregnancy. We excluded women without pre-pregnancy data for height and weight. Furthermore, we excluded women with excessively low (< 4500 kJ) or high (≥ 20 000 kJ) daily energy intake^(20)^.

### Measurements

Food and nutrient intake information during pregnancy were assessed using an FFQ validated for the Japanese population^(21,22)^. Detailed information on the FFQ has been provided elsewhere^(23)^. Briefly, pregnant women were administered a paper-based 172-items self-reported FFQ to assess dietary intake after pregnancy awareness. Women were asked to indicate how often they typically consumed specific foods, with nine options ranging from ‘never’ to ‘at least six times per day.’ The women chose the portion size for individual items by selecting small (50 % smaller than the standard portion size), medium (the same as the standard portion size) or large (50 % larger than the standard portion size). Food and nutrient intakes were calculated using an ad hoc computer algorithm based on the Standard Tables of Food Composition in Japan^(24)^ and then adjusted using the energy density method^(25)^. The correlation coefficients between FFQ and 12-day weighted dietary records ranged from 0·25 (seasonings) to 0·75 (fruits)^(21)^.

Information regarding folic acid supplementation was obtained through interviews. Dietary supplement intake was not included in the nutrient calculation because a reliable database for dietary supplements does not exist in Japan.

### Dietary patterns

We conducted a principal component analysis based on thirty food groups to identify specific dietary patterns. The foods were grouped according to categories listed in the Food Composition Table (5th) in Japan, and items with similar nutrient profiles or culinary applications were combined^(24)^ (online Supplementary Table 1).

Correlated food items were grouped as dietary patterns using principal component analysis. The principal component analysis can capture the population’s variation in the combination of food intake and is the most widely used method for deriving dietary patterns^(26)^. Dietary patterns were named based on the values of factor loadings, which indicated how much each food group correlated with each dietary pattern. Three dietary patterns were selected based on the scree plot for determining the breaking point of eigenvalues and interpretability^(15)^. Varimax rotation was used to maximise the sum of eigenvalues (the variance of the loading factors). Each woman’s score for each dietary pattern was calculated by summing the intake of food groups weighted by their factor loadings after energy-adjusted by the density method and standardisation. Food intake was log-transformed when the intake of food groups showed a skewed distribution.

Table 2 shows the results of the factor loading matrix for each dietary pattern. Furthermore, we conducted principal component analysis on two random halves of the data to confirm reproducibility^(27)^. The results of the factor loadings and component scores aligned well with our initial findings.

### Weight status

We obtained pre-pregnancy heights and weights from medical charts during prenatal visits to determine their BMI: < 17·0 kg/m^2^, severely or moderately underweight; 17·0–18·4 kg/m^2^, mildly underweight; 18·5–24·9 kg/m^2^, normal weight; 25·0–29·9 kg/m^2^, overweight and ≥ 30 kg/m^2^, obese^(28)^. Anthropometric data in medical charts were extracted by physicians, midwives, nurses and research coordinators in a standardised manner for JECS.

### Covariates

Covariates were chosen based on previous literature^(27,29)^. All covariates were obtained through the self-reported questionnaires. A woman’s age was categorised as < 25, 25–29, 30–34 or ≥ 35 years; parity as primiparous or multiparous; marital status as married or unmarried; annual household income as < 2 000 000, 2 000 000–3 999 999, 4 000 000–5 999 999 or ≥ 6 000 000 Japanese yen; the educational level as ≤ 12, 13–15 or ≥ 16 years and smoking status as ‘never,’ ‘stopped smoking before becoming aware of the pregnancy,’ ‘stopped smoking after becoming aware of the pregnancy’ or ‘continued smoking during pregnancy.’

### Statistical analyses

Food intake was energy-adjusted by density method. We calculated the mean and sd for the energy-adjusted intake of food groups across the quintile scores of three dietary patterns. Spearman’s correlation coefficients were also calculated to determine the correlations between dietary patterns and energy-adjusted nutrient intake.

Multivariate regression analysis was performed using the outcomes of dietary pattern scores. Pre-pregnancy BMI was considered an explanatory variable and adjusted for women’s age, parity, educational level, smoking status and trimester (the second or the third trimester)^(29)^. The models were mutually adjusted for the three dietary patterns. Because this study was a secondary analysis of data, we calculated the power of the analysis. A sample of 90 765 pregnant women would be sufficient to detect a 0·01 difference with more than 99·9 % power and an alpha of 0·05. All statistical analyses were performed using SAS version 9.4 (SAS Institute Inc.).

## Results

A total of 90 765 pregnant women were included in this study. The numbers (percentages) of women that were severely or moderately underweight, mildly underweight, normal weight, overweight and obese were 2463 (2·7 %), 12 105 (13·3 %), 66 640 (73·4 %), 7316 (8·1 %) and 2241 (2·5 %), respectively. Table 1 shows the characteristics of the women, grouped by pre-pregnancy weight status. Compared with normal-weight women, severely or moderately underweight women were younger, whereas those with obesity were older. Women with obesity and those who were severely or moderately underweight had lower income and lower education and continued to smoke during pregnancy compared with normal-weight women.

**Table 1:** Characteristics of women according to pre-pregnancy weight status

	Severely or moderately underweight (BMI: < 17·0 kg/m^2^, *n* 2463)		Mildly underweight (BMI: 17·0–18·4 kg/m^2^, *n* 12 105)		Normal weight (BMI: 18·5–24·9 kg/m^2^, *n* 66 640)		Overweight (BMI:25·0–24·9 kg/m^2^, *n* 7316)		Obese (BMI: ≥30 kg/m^2^, *n* 2241)	
	n	%	n	%	n	%	n	%	n	%
Age (years)										
<25	399	16·2	1517	12·5	6185	9·3	648	8·9	198	8·8
25–30	838	34·0	3814	31·5	18 578	27·9	1837	25·1	545	24·3
30–35	795	32·3	4226	34·9	23 926	35·9	2617	35·8	820	36·6
>35	431	17·5	2544	21·0	17 939	26·9	2212	30·2	677	30·2
Missing data	0	0·0	4	0·0	12	0·0	2	0·0	1	0·0
Parity										
Primiparity	847	34·4	3982	32·9	20 310	30·5	1872	25·6	549	24·5
Multiparity	1585	64·4	7976	65·9	45 642	68·5	5367	73·4	1668	74·4
Missing data	31	1·3	147	1·2	688	1·0	77	1·1	24	1·1
Marital status										
Married	2277	92·4	11 384	94·0	63 633	95·5	6967	95·2	2121	94·6
Not married	154	6·3	596	4·9	2457	3·7	286	3·9	93	4·1
Missing data	32	1·3	125	1·0	550	0·8	63	0·9	27	1·2
Household income (10 000 yen)										
<200	150	6·1	652	5·4	3015	4·5	513	7·0	204	9·1
200–<400	869	35·3	3876	32·0	20 704	31·1	2599	35·5	862	38·5
400–<600	673	27·3	3585	29·6	21 133	31·7	2192	30·0	658	29·4
≥ 600	524	21·3	3117	25·8	17 584	26·4	1515	20·7	366	16·3
Missing data	247	10·0	875	7·2	4204	6·3	497	6·8	151	6·7
Maternal education										
≤12 years	1015	41·2	4157	34·3	22 408	33·6	3184	43·5	1174	52·4
13–15 years	942	38·2	4996	41·3	28 627	43·0	3036	41·5	833	37·2
≥16 years	496	20·1	2918	24·1	15 378	23·1	1067	14·6	221	9·9
Missing data	10	0·4	34	0·3	227	0·3	29	0·4	13	0·6
Maternal smoking status										
Never smoked	1380	56·0	7215	59·6	39 064	58·6	3819	52·2	1030	46·0
Stopped smoking	900	36·5	4220	34·9	24 457	36·7	2985	40·8	1015	45·3
Continued to smoke during pregnancy	167	6·8	575	4·8	2625	3·9	435	6·0	162	7·2
Missing data	16	0·6	95	0·8	494	0·7	77	1·1	34	1·5
Folic acid supplement use										
Yes	908	36·9	4794	39·6	26 127	39·2	2485	34·0	691	30·8
No	1555	63·1	7311	60·4	40 513	60·8	4831	66·0	1550	69·2
Missing data	12	0·5	54	0·5	284	0·4	26	0·4	4	0·2

Three dietary patterns were identified using principal component analysis. Table 2 lists the factor loading matrices. The patterns were named according to the food groups with high loadings. For example, the first dietary pattern was named ‘vegetable and fruit,’ which had high loadings for green and white vegetables, potatoes, mushrooms, seaweed, fruits, fish, fat and oil and seasoning, but low white rice. The second dietary pattern was named ‘white rice and soy product’, which had high loadings for white rice, soya products and seasonings but low loadings for bread. The third dietary pattern was named ‘confectionery’, which had high loadings for confectionery and savory snack foods but low loadings for fresh and processed meat. These three principal components explained 18·9 % of the variance.

**Table 2:** Factor-loading matrix for three dietary patterns

	Vegetables and fruits	White rice and soya products	Confectionery	Kaiser–Meyer–Olkin
Well-milled rice	−0·63	0·53	−0·14	0·30
Unpolished rice	0·12	0·03	0	0·10
Bread	−0·02	−0·36	0·09	0·18
Noodles	0·15	−0·14	−0·05	0·12
Potatoes	0·57	0·06	−0·1	0·85
Nuts	0·15	−0·01	0·09	0·61
Soya products	0·17	0·7	0·1	0·45
Sugar	0·22	−0·02	0·09	0·36
Sweet confectionery	0·12	−0·22	0·59	0·44
Salty confectionery	0·11	−0·16	0·53	0·59
Fat and oil	0·32	−0·24	0·27	0·56
Fruit	0·49	−0·02	0·02	0·77
Green vegetables	0·55	0·17	−0·07	0·72
White vegetables	0·71	0·11	−0·17	0·79
Pickled vegetables	0·22	0·13	0·07	0·78
Mushrooms	0·52	0·1	−0·1	0·87
Seaweed	0·34	0·19	−0·01	0·88
Fruit and vegetable juice	0·03	−0·09	0·03	0·15
Green tea	−0·02	0·01	−0·06	0·56
Black tea	0·02	−0·06	−0·03	0·59
Coffee	−0·02	−0·06	0·02	0·14
Soft drinks	−0·08	−0·11	0·09	0·13
Fish	0·36	0·02	−0·11	0·59
Fresh meats	0·13	−0·07	−0·47	0·19
Processed meat	0·1	0·04	−0·31	0·61
Egg	0·05	−0·02	−0·07	0·14
Low fat milk	−0·01	−0·07	0·02	0·08
Full fat milk	−0·04	−0·15	0·1	0·11
Other dairy products	0·3	−0·12	−0·02	0·46
Seasonings	0·41	0·61	0·04	0·56
The number of food groups identified in each dietary pattern	9	4	4	–
Eigenvalues	2·94	1·75	0·99	–
Variance of explained (%)	9·7	5·4	3·9	–
Cumulative variance explained (%)	9·7	15·0	18·9	–

Definitions for the food groupings are provided in Supplementary Table 1.

Supplementary Table 2 shows the mean consumption according to each food group across the three dietary patterns. The ‘vegetable and fruit’ pattern was characterised by a high intake of green and white vegetables, potatoes, mushrooms, seaweed, fruits, fat and oil and seasoning but a low intake of white rice. The ‘white rice and soy product’ pattern was characterised by a high intake of white rice, soya products and seasonings but a low intake of bread. The ‘confectionery’ pattern had a high intake of confectionery and savory snack foods but a low intake of fresh and processed meat.

Supplementary Table 3 shows the correlation between women’s dietary patterns and nutrient intake. Specifically, the ‘vegetables and fruits’ pattern was correlated positively with the intake of fibre and most vitamins and minerals. The ‘white rice and soya products’ pattern was correlated positively with the intake of carbohydrates and negatively with the intake of saturated fat, Ca, vitamin B_2_ and vitamin E. The ‘confectionery’ pattern was correlated negatively with the intake of Ca, Zn and pantothenic acid.

Table 3 shows associations between women’s pre-pregnancy BMI and their dietary patterns. Women with obesity before pregnancy were significantly less likely to adhere to the ‘fruits and vegetables’ pattern (coefficient, –0·18; 95 % CI, –0·21, –0·14) and the ‘confectionery’ pattern (coefficient, –0·18; 95 % CI, –0·21, –0·14) and more likely to adhere to the ‘white rice and soy products’ pattern (coefficient, 0·08; 95 % CI, 0·04, 0·11) during pregnancy than normal pre-pregnancy weight. Women who were severely or moderately underweight were more likely to adhere to the ‘confectionery’ pattern (coefficient, 0·06; 95 % CI, 0·03, 0·09) than women with normal pre-pregnancy weight.

**Table 3:** Associations between women’ dietary patterns during pregnancy and BMI before pregnancy

	Vegetables and fruits			White rice and soya products			Confectionery		
	Coefficient	95 % CI	*P* value	Coefficient	95 % CI	*P* value	Coefficient	95 % CI	*P* value
BMI category									
Severely or moderately underweight	−0·01	–0·05, 0·02	0·3947	0·01	–0·02, 0·05	0·4374	0·06	0·03, 0·09	0·0002
Mild underweight	0·03	0·01, 0·05	0·0006	0·01	–0·01, 0·03	0·2903	0·02	0·01, 0·04	0·0037
Normal weight	Ref			Ref			Ref		
Overweight	−0·12	–0·14, −0·10	<0·0001	0·03	0·01, 0·05	0·0120	−0·09	–0·10, −0·07	<0·0001
Obesity	−0·18	–0·21, −0·14	<0·0001	0·08	0·04, 0·11	<0·0001	−0·18	–0·21, −0·14	<0·0001

Ref, reference.

Multiple regression analysis was performed after adjustment for age, parity, educational level, smoking status and trimester. All dietary pattern scores were mutually adjusted.

## Discussion

We found that obesity before pregnancy was associated with reduced adherence to ‘fruits and vegetables’ and ‘confectionery’ dietary patterns and increased adherence to ‘white rice and soy products’ and ‘confectionery’ dietary patterns during pregnancy. Additionally, a severely or moderately underweight status before pregnancy was associated with higher adherence to ‘confectionery’ dietary patterns during pregnancy.

Several studies have reported that women with obesity often consume fewer fruits and vegetables than their lean counterparts in non-pregnant women in Western and Asian countries^(16,30–32)^. However, few studies have investigated the relationship between pre-pregnancy weight status and food intake in pregnant women. A study in New Zealand showed that a health-conscious dietary pattern characterised by fruits, vegetables and whole grains was associated with a lower pre-pregnancy BMI^(33)^. In the present study, obesity before pregnancy was associated with lower adherence to the fruits and vegetable pattern during pregnancy; this finding is consistent with the aforementioned findings in pregnant and non-pregnant women^(30–32)^. Pregnant women with obesity are at risk of a low intake of fruits and vegetables. Therefore, dieticians and other health care professionals should inquire with obese pregnant women about the adequacy of intake of fruits and vegetables. They can then give dietary advice on how to ensure sufficient vitamin and mineral intake.

Our analyses also revealed that women with pre-pregnancy obesity adhered to a traditional Japanese diet characterised by high amounts of white rice and soya products and low quantities of bread and oils. Similarly, tangential investigations have demonstrated that high consumption of white rice and soya products elevates the risk of high BMI in young Japanese women^(32)^ and that eating white rice is linked to obesity across various Asian populations^(34,35)^. These findings from the earlier and present studies may be explained by the fact that white rice has a high glycaemic index and load, which stimulates insulin secretion and promotes weight gain, thereby resulting in obesity^(36)^.

Interestingly, our findings also demonstrated that underweight women were more likely to follow a ‘confectionery’ dietary pattern during pregnancy. A Japanese study showed that, compared to normal-weight women, underweight young women with a desire for thinness had sweet foods, composed of a higher percentage of their energy intake^(37)^. Another study indicated that women with anorexia nervosa, characterised by overestimation of weight and shape as well as self-starvation, preferred sweets taste and frequently consumed artificially sweetened foods^(38,39)^. These results may suggest that starvation physiologically enhances one’s desire for sweets and that underweight women strive to eat highly palatable food during pregnancy^(40)^. Another possible reason might be that pregnant underweight women may try to gain weight during pregnancy by the intake of palatable and less expensive food, such as confectionery. In contrast, overweight and obese women are less likely to follow a ‘confectionery’ dietary pattern during pregnancy. This may be because they are trying to avoid excessive weight gain during pregnancy.

The strengths of this study were the large sample size and the generalisability of the results to Japanese women. However, this study has several limitations. First, the dietary assessment was self-reported, which might have resulted in misreporting^(41,42)^. Overweight women might be likely to under-report their dietary intake, whereas underweight women might over-report their dietary intake^(43)^. This might have led to an underestimation of the differences in dietary patterns. Second, data on BMI before pregnancy were reported by women, which might have underestimated or overestimated their weight. However, categorising self-reported pre-pregnancy weight status has been proven to be valid^(44)^. Furthermore, the prevalence of women who were underweight in this study was not markedly different from that of the Japanese women of reproductive age in other Japanese studies^(45)^. Lastly, the FFQ has been validated for the Japanese population, but not specifically for pregnant women^(21,22)^.

Nutrient requirements increase during pregnancy. Our findings suggest that dietary advice should be according to weight status. In the present study, underweight women were more likely to have confectionery patterns, suggesting a high preference for sweets or palatable foods. Thus, dietary advice for underweight women should consider both food preferences and nutrient requirements. For overweight or obese women, dieticians and other healthcare professionals should ensure adequate intake of fruits and vegetables and advise against excessive consumption of white rice.

In conclusion, we found that maternal dietary patterns differed according to the pre-pregnancy weight status. Women with obesity or those who were severely or moderately underweight were more likely to consume the ‘confectionery’ pattern than normal weight women. These findings suggest that prenatal dietary advice is needed for pregnant women according to pre-pregnancy weight status.

## Supplementary material

For supplementary material accompanying this paper visit https://doi.org/10.1017/S1368980023000770. click here to view supplementary material
