# Supplementary material for: Association between pre-pregnancy weight status and dietary patterns during pregnancy: results from the Japan Environment and Children’s Study
Source: Public Health Nutr. 2023 May 2;26(9):1807–14. doi: 10.1017/S1368980023000770 (PMC10478052; doi:10.1017/S1368980023000770)
Supplement: Supplementary file 1 [file S1368980023000770sup001.docx]

# Supplementary Table 1. Food groups in the factor analysis

| Food group | Examples of food items |
| --- | --- |
| White rice | White rice |
| Unpolished rice | Barley rice, brown rice, and millet |
| Bread | Bread |
| Noodles | Buckwheat noodles, Chinese noodles, Japanese wheat noodles, Okinawan noodles, and pasta |
| Potatoes | Potato, sweet potato, taro, yam, and yam cake |
| Nuts | Peanut and sesame |
| Soybeans | Tofu and tofu products |
| Green vegetables | Bok choy, broccoli, carrot, Chinese chives, garland chrysanthemum, green asparagus, green onion, Japanese spinach, leaf mustard, mugwort, pumpkin, sweet pepper, tomato, turfed stone leek, snap peas, spinach, and swiss chard |
| White vegetables | Bean sprouts, bitter gourd, burdock, cabbage, Chinese cabbage, cucumber, eggplant, garlic, lettuce, onion, radish, and sponge gourd |
| Pickled vegetables | Pickled Chinese cabbage, pickled cucumber, and pickled eggplant, pickled green leaf, pickled plum, and pickled radish |
| Mushrooms | Enoki, Shiitake, and Shimeji |
| Seaweed | Wakame. hijiki and laver |
| Fruits | Apple, banana, grapes, Japanese persimmon, mandarin and other oranges, kiwifruit, mango, melon, watermelon, papaya, peach, pear, pineapple, and strawberry |
| Fruit and vegetable juice | Apple (100%), orange (100%), grapefruit (100%), and other types of fruit juices, and tomato and vegetable juice |
| Fish and shellfish | Salmon, tuna, horse mackerel/sardine, pacific saury/mackerel, yellow tail, cod/flatfish, sea bream, baby sardine, salmon roe, eel, squid, octopus, shrimp, clam, pond snail, fish cake, tube-shaped fish paste cake, fried fish cake, salted fillet, dried fish, and canned tuna |
| Fresh meat | Chicken, pork, and beef |
| Processed meat | Ham, sausage, bacon, and luncheon meat |
| Eggs | Egg |
| Full-fat milk | Full-fat milk |
| Low-fat milk | Low-fat milk |
| Other dairy products | Yogurt, cheese, and cream or creamer added to coffee |
| Green tea | Green tea |
| Black tea | Black tea |
| Coffee | Coffee |
| Soft drink | Soft drink |
| Confectionery | Cake, biscuits/cookies, chocolate, ice cream, and Japanese sweets |
| Savory snack foods | Snacks (potato chips and others) and rice crackers |
| Sugar | Honey, jam, marmalade, and sugar for coffee and tea |
| Oil and fat | Butter, vegetable oil (safflower, corn, soybean, rapeseed, olive, and other vegetable oil), and margarine |
| Seasonings | Salt for cooking, salad dressing, mayonnaise, Japanese brown sauce, and ketchup |

# Supplementary Table 2 food intake according to the dietary pattern

| **Food intake (g/1000kcal)** | **Dietary pattern score** | | | | | | | | | |
| --- | --- | --- | --- | --- | --- | --- | --- | --- | --- | --- |
|  | The lowest score group (Q1) | | Q2 | | Q3 | | Q4 | | The highest score group (Q5) | |
|  | Mean | (SD) | Mean | (SD) | Mean | (SD) | Mean | (SD) | Mean | (SD) |
| **Vegetable and fruits pattern** |  |  |  |  |  |  |  |  |  |  |
| White rice | 259.2 | (117.0) | 193.2 | (66.1) | 170.0 | (59.8) | 146.5 | (56.0) | 111.9 | (55.0) |
| Unpolished rice | 3.2 | (20.9) | 3.9 | (18.8) | 5.2 | (21.6) | 7.1 | (24.3) | 13.3 | (36.5) |
| Bread | 25.3 | (33.3) | 25.2 | (26.0) | 23.9 | (20.7) | 22.6 | (17.4) | 20.6 | (16.0) |
| Noodles | 53.5 | (42.3) | 62.2 | (38.4) | 65.9 | (37.9) | 68.5 | (38.3) | 67.6 | (39.4) |
| Potatoes | 6.5 | (6.1) | 10.6 | (7.0) | 13.1 | (7.8) | 16.0 | (8.8) | 21.7 | (13.5) |
| Nuts | 0.1 | (0.4) | 0.2 | (0.6) | 0.3 | (0.7) | 0.3 | (0.8) | 0.5 | (1.2) |
| Soy products | 70.0 | (82.9) | 78.7 | (66.2) | 87.0 | (67.2) | 95.3 | (68.7) | 111.6 | (76.1) |
| Sugar | 1.7 | (2.4) | 2.4 | (2.6) | 2.8 | (2.7) | 3.1 | (2.8) | 3.6 | (3.6) |
| Sweet confectionery | 26.7 | (55.3) | 35.0 | (43.9) | 37.7 | (36.9) | 40.1 | (36.1) | 42.3 | (46.3) |
| Salty confectionery | 4.7 | (11.3) | 6.1 | (8.5) | 6.8 | (8.7) | 7.3 | (8.2) | 7.7 | (10.4) |
| Fat and oil | 6.9 | (7.8) | 9.7 | (8.7) | 11.0 | (7.5) | 12.3 | (7.3) | 15.1 | (10.0) |
| Fruit | 23.4 | (32.6) | 37.4 | (37.4) | 47.2 | (43.1) | 58.3 | (49.6) | 80.4 | (67.6) |
| Green vegetables | 15.5 | (12.0) | 23.9 | (12.7) | 29.3 | (14.1) | 35.7 | (16.2) | 54.2 | (32.5) |
| White vegetables | 22.8 | (16.0) | 35.1 | (16.9) | 42.5 | (18.8) | 51.1 | (21.7) | 74.6 | (41.7) |
| Pickled vegetables | 2.3 | (4.6) | 3.4 | (5.3) | 4.1 | (6.2) | 5.1 | (7.7) | 8.0 | (14.3) |
| Mushrooms | 2.0 | (2.7) | 3.7 | (3.5) | 4.9 | (4.0) | 6.2 | (4.7) | 9.1 | (7.1) |
| Seaweed | 1.6 | (2.2) | 2.5 | (2.4) | 3.1 | (2.7) | 3.8 | (3.1) | 5.5 | (6.3) |
| Fruit and vegetable juice | 56.9 | (87.9) | 61.9 | (78.0) | 61.3 | (74.0) | 61.6 | (70.9) | 59.8 | (71.8) |
| Green tea | 119.9 | (245.4) | 105.0 | (181.6) | 100.7 | (176.7) | 99.2 | (167.2) | 101.7 | (164.7) |
| Black tea | 18.1 | (48.9) | 18.4 | (41.2) | 19.1 | (44.2) | 19.6 | (40.5) | 20.4 | (42.3) |
| Coffee | 43.7 | (86.5) | 43.5 | (71.4) | 40.7 | (63.2) | 38.9 | (59.0) | 37.3 | (56.4) |
| Soft drinks | 72.7 | (115.1) | 62.9 | (81.3) | 57.1 | (69.0) | 53.3 | (63.3) | 46.4 | (57.8) |
| Fish | 10.7 | (10.5) | 16.0 | (11.2) | 19.1 | (12.4) | 22.0 | (13.9) | 26.3 | (17.2) |
| Fresh meats | 22.4 | (22.7) | 21.8 | (14.9) | 22.0 | (14.8) | 21.7 | (14.1) | 20.7 | (16.0) |
| Processed meat | 3.7 | (5.0) | 3.9 | (3.9) | 4.0 | (3.8) | 4.0 | (3.8) | 3.9 | (3.9) |
| Egg | 15.3 | (19.8) | 17.0 | (17.0) | 16.8 | (14.9) | 16.6 | (13.9) | 16.4 | (13.1) |
| Low fat milk | 41.2 | (190.9) | 40.0 | (171.3) | 36.6 | (145.2) | 33.9 | (120.1) | 34.0 | (111.8) |
| Full fat milk | 127.8 | (327.5) | 139.4 | (295.5) | 118.8 | (229.6) | 107.5 | (180.1) | 96.9 | (170.7) |
| Other dairy products | 27.7 | (46.8) | 37.4 | (53.1) | 41.3 | (55.1) | 45.3 | (58.6) | 51.1 | (64.4) |
| Seasonings | 7.6 | (6.5) | 9.1 | (5.2) | 10.2 | (5.3) | 11.2 | (5.4) | 12.8 | (6.1) |
| **White rice and soy products** |  |  |  |  |  |  |  |  |  |  |
| White rice | 106.0 | (52.9) | 146.2 | (58.6) | 173.4 | (64.2) | 209.0 | (94.6) | 246.2 | (94.5) |
| Unpolished rice | 5.4 | (22.1) | 6.2 | (24.2) | 6.4 | (23.0) | 7.0 | (26.4) | 7.7 | (30.8) |
| Bread | 38.4 | (38.6) | 25.5 | (17.6) | 22.2 | (15.8) | 18.2 | (14.7) | 13.3 | (12.8) |
| Noodles | 69.4 | (48.4) | 69.7 | (39.9) | 66.1 | (36.9) | 59.8 | (35.6) | 52.6 | (33.0) |
| Potatoes | 11.3 | (9.2) | 13.6 | (9.7) | 14.3 | (10.2) | 14.3 | (10.4) | 14.5 | (11.8) |
| Nuts | 0.3 | (0.8) | 0.3 | (0.7) | 0.3 | (0.8) | 0.3 | (0.7) | 0.2 | (1.0) |
| Soy products | 34.8 | (26.4) | 54.5 | (33.4) | 72.8 | (39.8) | 99.0 | (50.4) | 181.4 | (91.3) |
| Sugar | 2.5 | (3.0) | 2.9 | (2.8) | 2.9 | (2.7) | 2.8 | (2.9) | 2.5 | (3.1) |
| Sweet confectionery | 53.5 | (64.6) | 41.8 | (37.6) | 35.1 | (33.5) | 28.8 | (25.2) | 22.5 | (44.9) |
| Salty confectionery | 9.0 | (13.9) | 7.6 | (8.8) | 6.4 | (7.3) | 5.3 | (6.6) | 4.3 | (8.7) |
| Fat and oil | 14.2 | (13.8) | 11.9 | (7.2) | 10.9 | (6.4) | 9.7 | (6.0) | 8.2 | (6.6) |
| Fruit | 48.1 | (59.4) | 50.7 | (51.2) | 50.9 | (49.4) | 49.3 | (48.4) | 47.7 | (47.7) |
| Green vegetables | 23.6 | (16.3) | 29.7 | (18.8) | 32.8 | (21.2) | 34.8 | (24.1) | 37.6 | (30.2) |
| White vegetables | 34.8 | (24.4) | 43.0 | (26.6) | 46.9 | (28.5) | 49.0 | (30.9) | 52.4 | (37.0) |
| Pickled vegetables | 3.0 | (5.0) | 3.9 | (6.1) | 4.5 | (6.9) | 5.0 | (8.4) | 6.5 | (13.5) |
| Mushrooms | 3.9 | (4.2) | 5.0 | (4.7) | 5.5 | (5.1) | 5.7 | (5.5) | 5.9 | (6.1) |
| Seaweed | 2.1 | (2.3) | 2.9 | (2.7) | 3.4 | (3.1) | 3.7 | (3.5) | 4.5 | (6.3) |
| Fruit and vegetable juice | 68.3 | (90.5) | 67.0 | (76.4) | 62.1 | (72.7) | 56.4 | (70.5) | 47.7 | (70.2) |
| Green tea | 103.2 | (194.0) | 104.9 | (187.6) | 104.3 | (179.9) | 104.5 | (185.8) | 109.4 | (200.2) |
| Black tea | 22.7 | (50.1) | 21.5 | (44.6) | 18.8 | (38.8) | 17.7 | (46.6) | 14.9 | (35.3) |
| Coffee | 47.0 | (78.0) | 44.4 | (68.6) | 41.1 | (64.9) | 37.0 | (62.3) | 34.7 | (65.4) |
| Soft drinks | 72.4 | (100.2) | 65.2 | (81.7) | 58.9 | (75.5) | 51.9 | (71.3) | 43.9 | (66.0) |
| Fish | 16.5 | (14.9) | 19.7 | (14.3) | 19.9 | (13.9) | 19.6 | (14.2) | 18.4 | (13.7) |
| Fresh meats | 23.4 | (20.7) | 23.1 | (15.7) | 22.2 | (16.8) | 20.6 | (14.6) | 19.3 | (15.2) |
| Processed meat | 3.6 | (4.4) | 3.9 | (4.0) | 4.0 | (3.9) | 3.9 | (3.9) | 4.0 | (4.3) |
| Egg | 16.5 | (19.2) | 16.7 | (16.1) | 16.9 | (15.9) | 16.3 | (14.4) | 15.7 | (13.4) |
| Low fat milk | 63.0 | (241.2) | 41.7 | (156.9) | 33.5 | (121.4) | 27.3 | (98.3) | 20.2 | (74.5) |
| Full fat milk | 205.3 | (409.6) | 130.0 | (242.2) | 103.4 | (189.2) | 85.7 | (151.4) | 66.0 | (114.3) |
| Other dairy products | 56.2 | (88.1) | 41.2 | (52.2) | 39.0 | (46.0) | 35.0 | (40.4) | 31.4 | (36.5) |
| Seasonings | 5.6 | (2.9) | 7.8 | (3.3) | 9.3 | (3.7) | 11.0 | (4.5) | 17.0 | (7.2) |
| **Confectionery pattern** |  |  |  |  |  |  |  |  |  |  |
| White rice | 199.2 | (83.8) | 187.0 | (78.0) | 176.8 | (74.4) | 163.3 | (73.0) | 154.5 | (121.6) |
| Unpolished rice | 5.9 | (23.6) | 6.8 | (25.1) | 7.0 | (25.9) | 6.9 | (25.7) | 6.2 | (27.0) |
| Bread | 20.0 | (17.4) | 22.3 | (17.6) | 23.0 | (19.1) | 24.3 | (23.5) | 28.0 | (35.0) |
| Noodles | 65.4 | (40.6) | 65.9 | (39.0) | 64.4 | (37.3) | 63.3 | (37.3) | 58.5 | (43.3) |
| Potatoes | 14.6 | (10.9) | 14.1 | (10.0) | 13.8 | (9.8) | 13.4 | (10.1) | 12.0 | (10.8) |
| Nuts | 0.2 | (0.4) | 0.2 | (0.6) | 0.3 | (0.6) | 0.3 | (0.8) | 0.4 | (1.3) |
| Soy products | 65.2 | (50.2) | 82.6 | (60.0) | 94.5 | (69.2) | 101.9 | (76.8) | 98.3 | (98.0) |
| Sugar | 2.1 | (2.2) | 2.6 | (2.4) | 2.8 | (2.6) | 3.0 | (2.9) | 3.2 | (4.0) |
| Sweet confectionery | 15.1 | (11.8) | 23.2 | (15.0) | 30.9 | (18.6) | 40.8 | (23.7) | 71.7 | (82.1) |
| Salty confectionery | 2.3 | (2.5) | 3.8 | (3.5) | 5.2 | (4.4) | 7.4 | (5.9) | 13.9 | (17.4) |
| Fat and oil | 8.6 | (4.2) | 9.9 | (4.8) | 10.6 | (5.4) | 11.4 | (6.3) | 14.5 | (15.9) |
| Fruit | 40.3 | (42.7) | 47.0 | (44.8) | 50.9 | (48.5) | 54.3 | (53.7) | 54.2 | (63.4) |
| Green vegetables | 33.6 | (22.3) | 32.8 | (21.4) | 32.3 | (21.5) | 31.6 | (22.7) | 28.3 | (26.9) |
| White vegetables | 50.0 | (29.2) | 46.9 | (27.6) | 45.7 | (28.2) | 44.3 | (30.5) | 39.2 | (35.0) |
| Pickled vegetables | 3.4 | (5.4) | 4.0 | (6.3) | 4.5 | (6.9) | 5.2 | (9.0) | 5.9 | (13.0) |
| Mushrooms | 5.9 | (5.8) | 5.6 | (5.2) | 5.3 | (5.0) | 4.9 | (4.7) | 4.3 | (5.2) |
| Seaweed | 3.2 | (3.5) | 3.4 | (3.3) | 3.4 | (3.3) | 3.4 | (3.5) | 3.2 | (5.5) |
| Fruit and vegetable juice | 53.7 | (73.9) | 58.8 | (73.8) | 61.5 | (73.4) | 63.7 | (77.0) | 63.8 | (84.8) |
| Green tea | 139.8 | (263.5) | 107.8 | (178.7) | 97.7 | (170.1) | 92.6 | (150.8) | 88.6 | (158.3) |
| Black tea | 22.3 | (61.8) | 18.6 | (37.2) | 18.6 | (36.4) | 18.2 | (36.5) | 17.8 | (39.9) |
| Coffee | 37.7 | (69.1) | 39.3 | (64.7) | 41.6 | (66.0) | 43.5 | (68.2) | 42.2 | (72.5) |
| Soft drinks | 43.8 | (60.9) | 52.6 | (67.0) | 57.9 | (71.8) | 64.8 | (82.4) | 73.2 | (108.2) |
| Fish | 21.2 | (16.1) | 20.3 | (14.4) | 19.2 | (13.3) | 18.2 | (13.1) | 15.1 | (13.5) |
| Fresh meats | 37.1 | (23.8) | 25.0 | (12.6) | 20.3 | (11.4) | 16.1 | (9.0) | 10.1 | (7.8) |
| Processed meat | 6.5 | (6.0) | 4.3 | (3.7) | 3.6 | (3.1) | 3.0 | (2.8) | 2.0 | (2.5) |
| Egg | 18.5 | (17.4) | 17.1 | (15.1) | 16.7 | (15.2) | 15.9 | (15.2) | 14.0 | (16.4) |
| Low fat milk | 21.8 | (63.9) | 27.5 | (89.5) | 35.4 | (131.7) | 41.9 | (164.6) | 59.0 | (237.6) |
| Full fat milk | 49.7 | (70.3) | 76.2 | (104.0) | 100.1 | (147.2) | 138.8 | (240.3) | 225.5 | (442.8) |
| Other dairy products | 35.4 | (42.1) | 38.7 | (45.9) | 40.8 | (54.4) | 44.0 | (64.3) | 43.8 | (70.0) |
| Seasonings | 8.4 | (4.5) | 9.8 | (5.0) | 10.7 | (5.6) | 11.2 | (6.2) | 10.6 | (7.7) |

Q1, the first quintile of scores for dietary pattern; Q2, the second quintile of scores for dietary pattern; Q3, the third quintile of scores for dietary pattern; Q4, the fourth quintile of scores for dietary pattern; Q5, the fifth quintile of scores for dietary pattern; SD, standard deviation

**Supplementary Table 2**. Correlations between women’s dietary patterns and nutrient intakes

|  | Vegetables and fruits | White rice and soy products | Confectionery |
| --- | --- | --- | --- |
| Protein | 0.35 | -0.20 | -0.25 |
| Total fat | 0.24 | -0.37 | -0.05 |
| n–3 PUFA | 0.24 | -0.26 | 0.03 |
| n–6 PUFA | 0.51 | -0.03 | 0.11 |
| Saturated fatty acid | 0.03 | -0.50 | -0.13 |
| Carbohydrate | -0.23 | 0.32 | 0.11 |
| Dietary fiber | 0.81 | -0.05 | -0.02 |
| Calcium | 0.28 | -0.43 | -0.34 |
| Magnesium | 0.64 | -0.08 | -0.15 |
| Iron | 0.61 | 0.27 | 0.02 |
| Zinc | 0.16 | 0.07 | -0.43 |
| Vitamin A | 0.46 | -0.25 | -0.22 |
| Vitamin B_1_ | 0.50 | -0.15 | -0.13 |
| Vitamin B_2_ | 0.27 | -0.33 | -0.30 |
| Niacin | 0.40 | 0.05 | 0.12 |
| Vitamin B_6_ | 0.69 | 0.03 | -0.17 |
| Vitamin B_12_ | 0.32 | -0.12 | 0.00 |
| Folate | 0.69 | -0.03 | -0.14 |
| Pantothenic acid | 0.39 | -0.26 | -0.39 |
| Vitamin C | 0.61 | -0.07 | 0.04 |
| Vitamin D | 0.36 | -0.05 | 0.00 |
| Vitamin E | 0.66 | -0.23 | 0.02 |
| Vitamin K | 0.59 | 0.10 | -0.08 |
| Salt equivalent | 0.68 | 0.15 | 0.07 |

Abbreviation: PUFA, polyunsaturated fatty acids
